# The biosynthetic gene cluster for the cyanogenic glucoside dhurrin in *Sorghum bicolor* contains its co-expressed vacuolar MATE transporter

**DOI:** 10.1038/srep37079

**Published:** 2016-11-14

**Authors:** Behrooz Darbani, Mohammed Saddik Motawia, Carl Erik Olsen, Hussam H. Nour-Eldin, Birger Lindberg Møller, Fred Rook

**Affiliations:** 1 Plant Biochemistry Laboratory, Department of Plant and Environmental Sciences, University of Copenhagen, Thorvaldsensvej 40, 1871 Frederiksberg, Denmark; 2 VILLUM Research Center for Plant Plasticity, University of Copenhagen, Denmark; 3 Plant Molecular Biology, Department of Plant and Environmental Sciences, University of Copenhagen, Thorvaldsensvej 40, 1871 Frederiksberg, Denmark

## Abstract

Genomic gene clusters for the biosynthesis of chemical defence compounds are increasingly identified in plant genomes. We previously reported the independent evolution of biosynthetic gene clusters for cyanogenic glucoside biosynthesis in three plant lineages. Here we report that the gene cluster for the cyanogenic glucoside dhurrin in *Sorghum bicolor* additionally contains a gene, *SbMATE2*, encoding a transporter of the multidrug and toxic compound extrusion (MATE) family, which is co-expressed with the biosynthetic genes. The predicted localisation of *SbMATE2* to the vacuolar membrane was demonstrated experimentally by transient expression of a SbMATE2-YFP fusion protein and confocal microscopy. Transport studies in *Xenopus laevis* oocytes demonstrate that SbMATE2 is able to transport dhurrin. In addition, SbMATE2 was able to transport non-endogenous cyanogenic glucosides, but not the anthocyanin cyanidin 3-O-glucoside or the glucosinolate indol-3-yl-methyl glucosinolate. The genomic co-localisation of a transporter gene with the biosynthetic genes producing the transported compound is discussed in relation to the role self-toxicity of chemical defence compounds may play in the formation of gene clusters.

Plants produce a wide variety of chemical defence compounds that provide protection against herbivores and pathogens. A particular plant species or genus is characterised by the presence of a subset of such defence compounds. Considerable inter- and intraspecific variation is thought to result from various trades-offs, such as between growth and defence, or between competing defence strategies in a varying ecological context^1^,^2^. Besides constraints on the allocation of resources, chemical defence also carries the risk of self-toxicity as a metabolic cost. One specific class of chemical defence compounds are the cyanogenic glucosides, which occur widely in the plant kingdom^3^. These compounds are glucosides of amino acid derived α-hydroxynitriles, and part of a two-component chemical defence system. Hydrolysis of cyanogenic glucosides by a specific β-glucosidase following tissue disruption, for instance by chewing insects, releases the chemically unstable α-hydroxynitrile, which upon dissociation gives rise to the formation of toxic hydrogen cyanide. The cyanogenic glucoside dhurrin is the main chemical defence compound in *Sorghum bicolor*, an important cereal crop used for food and feed^4^. Dhurrin is derived from the amino acid tyrosine by the sequential action of two cytochrome P450 enzymes, named CYP79A1 (Sobic.001G012300) and CYP71E1 (Sobic.001G012200), and the glucosylation and stabilisation of the produced α-hydroxynitrile (cyanohydrin) intermediate by the UDP-glucosyltransferase UGT85B1 (Sobic.001G012400)^5^. As it involves two membrane anchored cytochrome P450 enzymes, dhurrin biosynthesis is thought to take place at the cytosolic surface of the endoplasmic reticulum (ER). The glucosylated compound, which is labile in non-acidic environments due to the ionization of the hydroxyl group on the benzene ring^6^, is stably stored in the acidic vacuolar compartment but the mechanism of its intracellular transport from the ER to the vacuole is unknown^7^.

The biosynthetic pathways for cyanogenic glucosides have also been elucidated in dicot plant species such as cassava (*Manihot esculenta*) and the model legume *Lotus japonicus*. Gene identification in *L. japonicus* was helped by the fact that the biosynthetic genes were found to be co-localised in the same genome region, and in this species the second step was catalysed by a member of the CYP736 gene family^8^. The biosynthetic genes in cassava and sorghum were also found to be organised in a gene cluster, but the three clusters are thought to have evolved independently. This remarkable genomic co-localisation of non-homologous genes encoding biosynthetic enzymes in the same metabolic pathway has also been observed for other classes of plant chemical defence compounds such as terpenoids^9^,^10^,^11^, benzoxazinoids^12^, and alkaloids^13^,^14^. These clusters are proposed to promote the co-inheritance of beneficially interacting alleles and to additionally facilitate the co-expression of the biosynthetic genes by regulation at the chromatin level^11^,^15^. An important driver for gene cluster formation and maintenance, via selection for reduced recombination between the interacting genes, is thought to be the fact that incompletely inherited biosynthetic pathways may result in the release of toxic intermediates causing self-toxicity^15^,^16^.

Membrane transport is increasingly recognised as an important component of plant specialised metabolism and bioengineering approaches, but the number of characterised transporters remains limited^17^. Members of the large multidrug and toxic compound extrusion (MATE) gene family are found in both prokaryotes and eukaryotes, and transport a wide range of compounds^18^. In plants they have been shown to transport xenobiotic compounds, organic acids^19^, plant hormones, and secondary metabolites such as anthocyanins and other flavonoids^20^,^21^,^22^, and the alkaloid nicotine^23^,^24^. Here we report that the biosynthetic gene cluster for dhurrin additionally includes a gene encoding a tonoplast localised MATE transporter for dhurrin uptake, demonstrating that the analysis of plant gene clusters can contribute to transporter identification.

## Results and Discussion

### The dhurrin gene cluster

**Figure 1:**
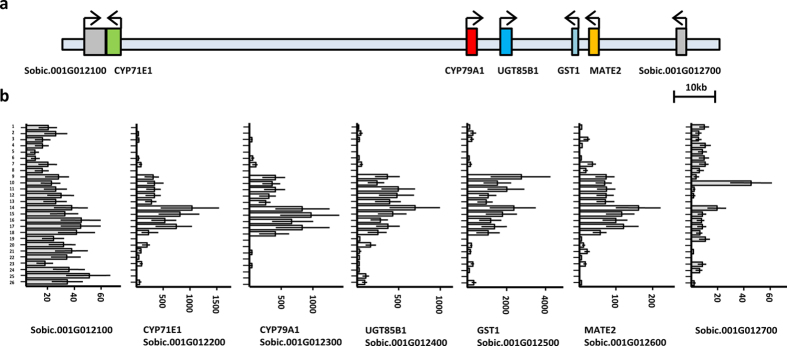
The dhurrin gene cluster in sorghum contains *SbMATE2* and *SbGST1*, which are co-expressed with the biosynthetic genes. (**a**) genomic organisation of the dhurrin gene cluster. (**b**) RNA-Seq expression profiles for the dhurrin biosynthetic genes *CYP79A1*, *CYP71E1*, and *UGT85B1*, and for *SbGST1* and *SbMATE2*, and two flanking genes. FPKM values (Fragments Per Kilobase Million) are indicated, for treatment details see Supplementary Table 1 (adapted from the MOROKOSHI transcriptome database).

Analysis of the genomic region surrounding the dhurrin biosynthetic gene cluster in sorghum, revealed the presence of genes encoding a MATE transporter (Sobic.001G012600) we have named *SbMATE2*, and a glutathione *S*-transferase (GST) named *SbGST1* (Sobic.001G012500) of the plant specific phi subfamily (Fig. 1a). Additional support for the involvement of these two genes in dhurrin metabolism was their co-expression with the biosynthetic genes, as revealed by searching the MOROKOSHI sorghum transcriptome database containing publically available RNA-seq data^25^. The genes showing the highest co-expression with *CYP79A1*, encoding the first enzyme of the dhurrin biosynthetic pathway, were *CYP71E1*, immediately followed by *SbMATE2* (Fig. 1b, Supplementary Table 1). Co-expression with *CYP79A1* was additionally observed for the *SbGST1* and the *UGT85B1* genes, which showed the highest level of co-expression with each other. High relative expression of all genes was observed in shoots of 9-day old seedlings (Fig. 1b, condition 16), which was enhanced by abscisic acid and osmotic stress treatments (conditions 14 and 15, respectively)^26^,^27^. Co-expression studies have resulted in transporter identification, as was reported for several MATE vacuolar nicotine transporters in *Nicotiana tabacum*^23^,^24^. Moreover, the co-expression of clustered biosynthetic genes from the same pathway was reported for the synthesis of the triterpene thalianol in *Arabidopsis thaliana*, and such tight coordinated regulation is suggested to prevent the accumulation of deleterious biosynthetic intermediates^11^. This toxicity argument also applies to the labile and reactive intermediates of the dhurrin pathway, and to dhurrin itself given its labile and reactive nature at cytoplasmic pH^6^. The coordinated expression of genes in transport or storage with those of the biosynthesis pathway, is similarly proposed to reduce or prevent the self-toxic effects of the metabolites produced.

GSTs are well known for conjugating the tripeptide glutathione to endogenous toxic products and xenobiotic compounds, but also for non-enzymatic roles as carrier proteins for endogenous reactive molecules such as porphyrins and anthocyanins^28^,^29^,^30^. Like in the biosynthesis of cyanogenic glucosides, anthocyanins are produced by cytochrome P450 enzymes on the cytoplasmic surface of the endoplasmic reticulum and it is of particular interest to note that anthocyanin transport to the vacuole requires the action of both a GST and a MATE transporter^31^. This is for instance demonstrated by the transparent testa mutants in Arabidopsis, where the *TT19* gene encodes a GST, and where *TT12* encodes a MATE transporter^21^,^32^. The precise mechanism of anthocyanin transport is the subject of much debate, may involve vesicle mediated trafficking, and was suggested to be related to the removal of toxic compounds from the cytoplasm^33^. Although similar mechanisms may have been recruited in the case of dhurrin, a potential role for SbGST1 in dhurrin metabolism remains to be established and could be indirect, such as in dealing with the cellular effects of dhurrin self-toxicity.

### SbMATE2 is localised to the vacuolar membrane

**Figure 2:**
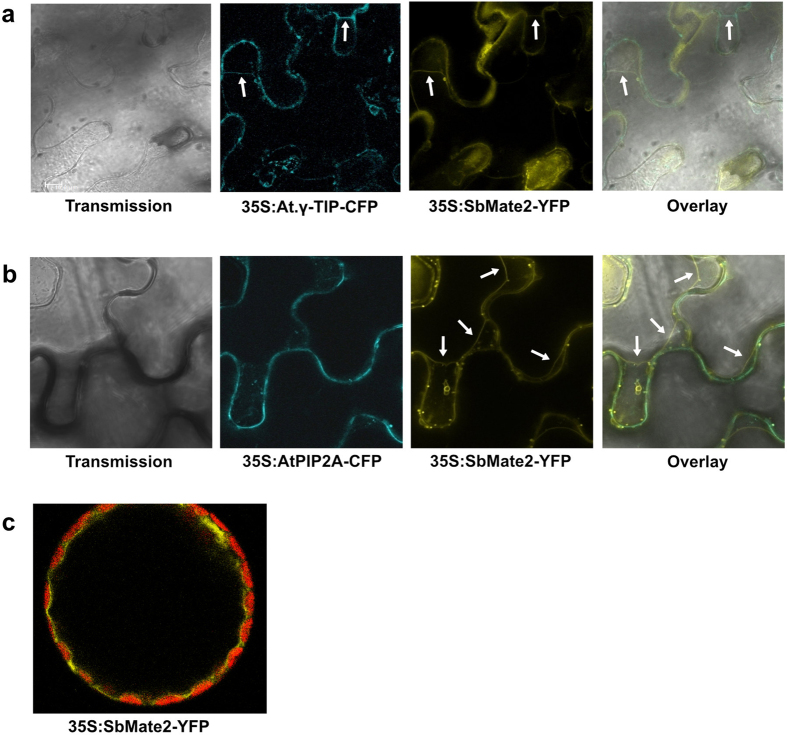
Tonoplast localisation of the SbMATE2-YFP fusion protein in epidermal cells and protoplasts of *Nicotiana benthamiana*. A SbMATE2-YFP fusion protein under the control of the 35S-CaMV promoter was transiently expressed in *N. benthamiana* using Agrobacterium infiltration. The SbMATE2-YFP construct was coinfiltrated with expression constructs for either: (**a**) At.γ-TIP-CFP, a marker for the vacuolar membrane, or (**b**) AtPIP2A-CFP, a marker for the plasma membrane^38^. The localisation of the fusion proteins was visualised using confocal microscopy, and the SbMATE-YFP signal co-localised with that of γ-TIP-CIP, showing a tonoplast localisation. Arrows indicate positions where the tonoplast is not adjacent to the plasma membrane. (**c**) Localisation of SbMATE2-YFP (in yellow) to the vacuolar membrane in isolated *N. benthamiana* protoplasts. Chloroplasts situated between the vacuolar and plasma membranes are visualised by their autofluorescence (in red).

The sequence of the *SbMATE2* transporter gene was experimentally verified by cDNA cloning from seedlings and shown to contain two small introns, 135 bp and 97 bp in length respectively, positioned in the C-terminal half of the protein coding region. The transcript encodes a 498 amino acid polypeptide predicted by the Phyre2 web portal for protein modelling to show the topology of the twelve transmembrane helixes typical for prokaryotic and plant MATE transporters^34^,^35^,^36^ (Supplemental Fig. 1). The structural model based on the NorM transporter from *Vibrio cholerae*, and an additional amino acid sequence alignment that includes mammalian MATE transporters, indicated that SbMATE2 contains conserved amino acids that are part of the cation-binding motif reported for NorM-VC^35^,^37^ (Supplemental Fig. 2). The functionality of a predicted N-terminal tonoplast targeting signal was experimentally investigated by transiently expressing a SbMATE2-YFP fusion protein in *Nicotiana benthamiana* followed by confocal microscopy. Co-expression of SbMATE2-YFP with either one of two aquaporin based organelle specific markers was used to distinguish between the tonoplast and plasma membrane^38^. SbMATE2-YFP co-localised with the vacuolar membrane marker γ-TIP-CFP, a C-terminal fusion of CFP to full-length γ-TIP, as both signals were observed at positions where the tonoplast was not localised directly adjacent to the cell wall (Fig. 2a). In contrast, the AtPIP2A-CFP marker, consisting of the CFP fused to the plasma membrane aquaporin AtPIP2A, followed the cellular outline precisely (Fig. 2b). Tobacco protoplasts expressing the *SbMATE2-YFP* construct were used to further exclude a plasma membrane localisation of SbMATE2. Confocal microscopy showed that the SbMATE2-YFP fusion protein was only localised to the vacuolar membrane as it folds around the chloroplasts on the side internal to the cell (Fig. 2c).

### SbMATE2 transports cyanogenic glucosides

**Figure 3:**
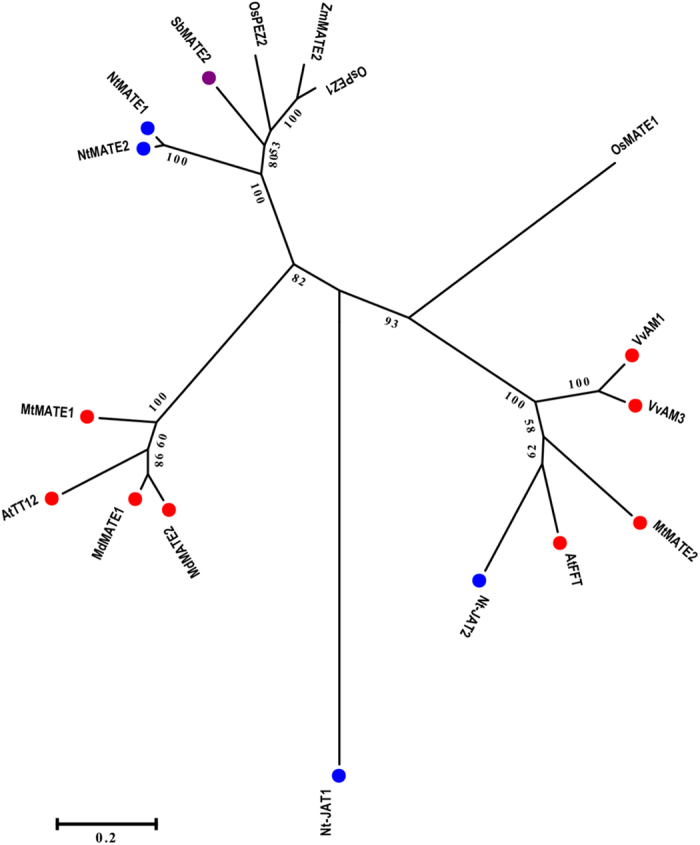
In a phylogenetic analysis SbMATE2 is part of a clade containing MATE transporters for flavonoids and alkaloids. A molecular phylogenetic analysis was performed using the Maximum Likelihood method based on the Jones-Taylor-Thornton (JTT) matrix-based model for amino acid sequences. Branch lengths are measured in the number of substitutions per site, and positions containing gaps were eliminated. Bootstrap values (1000x) are indicated at branch points. Analyses were conducted using the MEGA5 software package^47^. The amino sequences used in the analysis were: SbMATE2 (*Sorghum bicolor*, Sobic.001G012600), OsPEZ1 (*Oryza sativa*, Os03g37490), OsPEZ2 (*O. sativa*, Os03g0572900), OsMATE1 (*O. sativa*, Os03g08900), TT12 (*Arabidopsis thaliana*, At3g59030), FFT (*A. thaliana*, At4g25640), MdMATE1 (*Malus domestica*, GU64954), MdMATE2 (*M. domestica*, GU064956), MtMATE1 (*Medicago truncatula*, FJ858726), MtMATE2 (*M. truncatula*, HM856605), VvAM1 (*Vitis vinifera*, Fj264202), VvAM3 (*V. vinifera*, FJ264203), NtMATE1 (*Nicotiana tabacum*, AB286961), NtMATE2 (*N. tabacum*, AB286962), Nt-JAT1 (*N. tabacum*, AM991692), Nt-JAT2 (*N. tabacum*, AB922128), and ZmMATE2 (*Zea mays*, FJ873684). Coloured circles represent the transported compound classes: red = flavonoids, blue = alkaloids, purple = hydroxynitrile glucosides.

Phylogenetic analysis placed SbMATE2 in what Shitan *et al.* designated as clade I, consisting of MATE transporters that are functionally characterised (Fig. 3)^39^. Most of the MATE transporters in this clade function in the accumulation of plant specialised metabolites such as flavonoids and alkaloids, perhaps also a reflection of the experimental interest in transporters for these compounds. The clade includes the seed coat expressed vacuolar anthocyanin transporter AtTT12^21^,^40^, MtMATE1 and MtMATE2 from *Medicago truncatula* transporting flavonoid glycosides (and in the case of MtMATE2 also flavonoid glycoside malonates)^20^, VvAM1 and VvAM3 from *Vitis vinifera* transporting acylated anthocyanins^22^, and the tobacco NtMATE1, NtMATE2, and Nt-JAT2 transporters for the vacuolar sequestration of nicotine in leaves or roots of *Nicotiana tabacum*^23^,^24^.

**Figure 4:**
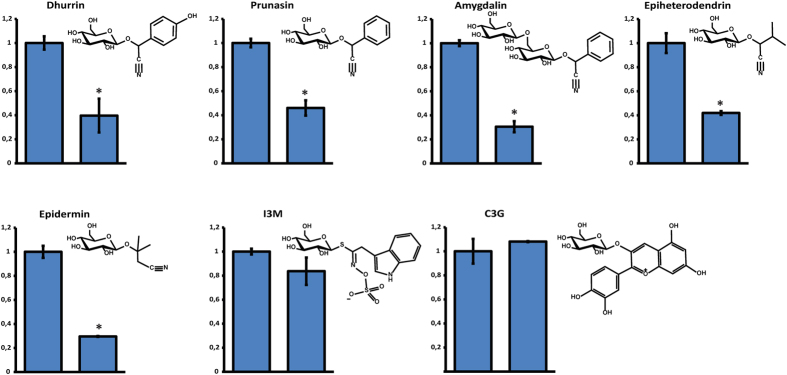
SbMATE2 exports dhurrin and other hydroxynitrile glucosides from *X. Laevis* oocytes, but not cyanidin 3-O-glucoside. The bars show the relative content of the indicated plant specialised metabolites in *SbMATE2* expressing (right bars) and non-expressing control (left bars) oocytes 90 min after injection of the respective compounds into the oocytes (means, ±s.e., statistical significant differences to control oocytes are indicated *p < 0.05). Each of the compound solutions was injected into 25–30 oocytes to an estimated internal concentration of 100 μM. Following incubation, oocytes were analysed in triplicates consisting of 7–10 oocytes. The compounds shown are the cyanogenic glucosides dhurrin, prunasin, amygdalin, epiheterodendrin, the non-cyanogenic β-hydroxynitrile glucoside epidermin, indol-3-yl-methyl glucosinolate (I3M), and the anthocyanin cyanidin 3-O-glucoside (C3G).

The possible role of SbMATE2 in dhurrin transport was studied by export experiments conducted in *Xenopus laevis* oocytes. Following injection of dhurrin and a 90 min incubation, SbMATE2 expressing oocytes showed an approximate 60% reduction in dhurrin content in comparison with oocytes not expressing SbMATE2, indicating dhurrin transport activity (Fig. 4). The SbMATE2 transporter was additionally able to transport the structurally related aromatic cyanogenic glucosides prunasin and the diglucoside amygdalin, the leucine derived cyanogenic glucoside epiheterodendrin and the non-cyanogenic β-hydroxynitrile glucoside epidermin (Fig. 4). This indicates that SbMATE2 shows a broad tolerance for accepting structurally varying hydroxynitrile glucoside compounds. We additionally tested if the transporter was able to transport cyanidin 3-O-glucoside (C3G), the anthocyanin substrate for AtTT12, and indol-3-yl-methyl glucosinolate (I3M), representing the glucosinolate class of compounds considered to be chemically and biosynthetically related to cyanogenic glucosides, but neither compound was transported in significant levels.

Our results demonstrate the presence of a non-biosynthetic component, the *SbMATE2* gene encoding a vacuolar transporter for dhurrin, in the gene cluster for a plant chemical defence pathway. Its inclusion in the dhurrin biosynthetic gene cluster is consistent with ideas that selection for reduced recombination between beneficially interacting alleles leads to gene cluster formation^15^,^16^,^41^. Such selection is proposed to result from antagonistic selection pressures, such as the benefits maintaining a functional pathway provides in specific ecological context, e.g. the presence of non-adapted herbivores, against the trade-off costs associated with it^1^,^15^. One of the negative cost associated with the production of chemical defence metabolites is the possibility of self-toxicity. Co-inheritance of a co-adapted gene set is thought to provide protection against the self-toxic biochemical nature of many chemical defence compounds or their pathway intermediates^15^,^16^. In the case of dhurrin the transport of this pH-dependent unstable cyanogenic glucoside from its cytoplasmic site of production to the acidic vacuole likely contributes to reducing self-toxicity. We previously also reported independently evolved biosynthetic gene clusters for cyanogenic glucosides in cassava and Lotus^8^. The main cyanogenic compounds produced by these species are linamarin and lotaustralin, respectively, which are more stable than dhurrin. In the most recent version of the cassava genome (*Manihot esculenta* v6.1), a MATE encoding gene named Manes.12G129000.1, which is not orthologous to *SbMATE2*, is present at a distance of about 325 kb of the described gene cluster. This physical linkage results in a high level of co-inheritance of Manes.12G129000.1 with the biosynthetic genes, but its physiological role is presently uncharacterised. The incomplete draft of the *Lotus japonicus* genome does not contain a transporter gene on the sequence contig that contains the biosynthetic gene cluster, but genetics has positioned at least one additional biosynthetic gene in hydroxynitrile glucoside metabolism in the vicinity of the gene cluster^8^. Eukaryotic biosynthetic gene clusters have been studied more extensively in fungi, and it is of interest to note that the inclusion of transporters is not uncommon in fungal gene clusters. The *TRI12* gene in *Fusarium sporotrichioides* is part of the biosynthetic gene cluster for terpene-derived trichothecene mycotoxins and encodes a trichothecene efflux pump^42^. Its disruption results in reduced growth and reduced levels of trichothecene production. A clear role in self-protection was reported for the *TOXA* gene in the fungal pathogen *Cochliobolus carbonum*, encoding an HC-toxin efflux pump essential for strains producing this toxic cyclic tetrapeptide^43^. Fungi also contain metabolic gene clusters which provide nutritional benefits under certain ecological conditions. For example, the *DAL* cluster in *Saccharomyces cerevisiae* allows the use of allantoin, a degradation product of purines, as a nitrogen source instead of urate, providing an advantage in oxygen-poor natural environments^44^. Apart from the catabolic genes, the *DAL* cluster also contains the *DAL4* gene encoding an allantoin permease. Given these examples from fungi, it can be expected that the future detailed analysis of genomic regions containing gene clusters for plant specialised metabolites will contribute to the identification of additional non-biosynthetic pathway components such as regulators or transporters.

## Methods

### Plant material, cDNA isolation and expression constructs

Total RNA was extracted from 3-day old etiolated seedlings of *Sorghum bicolor*. Following cDNA synthesis, a full length cDNA clone of *SbMATE2* was amplified, cloned using the Zero Blunt TOPO PCR Cloning Kit (Invitrogen), and its sequence was verified. For localisation studies, the *SbMATE2* protein coding region was amplified from the cDNA clone and fused in frame to *YFP* using USER cloning as described^45^. The *SbMATE2-YFP* construct, under control of the 35S-CaMV promoter, was transformed to *Agrobacterium tumefaciens* strain AGL1. For oocyte expression the *SbMATE2* coding region was cloned downstream of the T7 promoter in the USER compatible *Xenopus* expression vector pNB1u and linear template for *in vitro* transcription was generated by PCR. Further details can be found in the Supplementary information.

### Transient expression and confocal microscopy

Transient expression in tobacco was performed by *Agrobacterium* infiltration of *Nicotiana benthamiana* leaves. Visualisation of the fluorescent protein fusions in epidermal cells or isolated mesophyll protoplasts was carried out using a Leica TCS SP5-II confocal microscope. Excitation/emission wavelengths were 515/525–535 nm for YFP and 435/500–510 nm for CFP. The excitation/emission wavelengths for capturing chlorophyll autofluorescence were 544/660–690 nm.

### Oocyte transport assays

Oocytes from *Xenopus laevis* were obtained from EcoCyte Bioscience (Castrop-Rauxel, Germany). Capped cRNA of *SbMATE2* was synthesized using the mMESSAGE mMACHINE^®^ T7 Transcription Kit (ThermoFisher). For expression in oocytes, 25 ng of *in vitro* produced cRNA for the SbMATE2 transporter was injected into oocytes 4 days prior to performing transport assays essentially as described previously^45^. Assuming an oocyte volume of ~1 μL, 50 nL of 2 mM compound stock solutions were injected to obtain estimated internal concentrations of 100 μM. Using the same needle each compound was injected into 25–30 oocytes expressing *SbMATE2* and 25–30 control (non-expressing) oocytes. Following two washing steps, each batch of 25–30 oocytes was incubated for 90 min in 500 μL Kulori buffer at pH 5. After incubation, all intact oocytes were washed four times in ice-cold Kulori buffer pH 5 and 7–10 oocytes were extracted in triplicate in 50% MeOH as described previously^4^. Extracts were analysed by LC-MS. Statistical significant differences between the means of SbMATE2 expressing and control oocytes were calculated using a *t*-test and GraphPad Software (www.graphpad.com).

### Chemicals and LC-MS analysis

Amygdalin and cyanidin 3-O-glucoside were obtained from Sigma-Aldrich and indol-3-yl-methyl glucosinolate from Cfm Oskar Tropitzch GmbH. Dhurrin, prunasin, epiheterodendrin and epidermin were chemically synthesized^46^. LC-MS analysis was performed using a Zorbax SB-C18 column on an Agilent 1100 Series LC coupled to a Bruker HCT-Ultra ion trap mass spectrometer as described previously^8^. Compounds were localised in extracted ion chromatograms as sodium adduct ions: dhurrin (m/z 334), prunasin (m/z 318), amygdalin (m/z 480), epidermin (m/z 284), epiheterodendrin (m/z 284), cyanidin 3-O-glucoside (m/z 449), indol-3-yl-methyl glucosinolate (m/z 493). Relative quantification was based on peak area using Bruker-DataAnalysis 4.0 (Bruker Daltonik).

## Additional Information

**How to cite this article**: Darbani, B. *et al.* The biosynthetic gene cluster for the cyanogenic glucoside dhurrin in *Sorghum bicolor* contains its co-expressed vacuolar MATE transporter. *Sci. Rep.* **6**, 37079; doi: 10.1038/srep37079 (2016).

**Publisher's note:** Springer Nature remains neutral with regard to jurisdictional claims in published maps and institutional affiliations.

## Supplementary Material

Supplementary Information
