# Supplementary material for: The biosynthetic gene cluster for the cyanogenic glucoside dhurrin in Sorghum bicolor contains its co-expressed vacuolar MATE transporter
Source: Sci Rep. 2016 Nov 14;6:37079. doi: 10.1038/srep37079 (PMC5107947; doi:10.1038/srep37079)
Supplement: Supplementary Information [file srep37079-s1.pdf]

Supplementary information to:

The biosynthetic gene cluster for the cyanogenic glucoside dhurrin in *Sorghum bicolor* contains its co-expressed vacuolar MATE transporter

Behrooz Darbani, Mohammed Saddik Motawia, Carl Erik Olsen, Hussam H. Nour-Eldin, Birger Lindberg Møller, Fred Rook

Supplementary methods

Gene cloning and expression constructs

cDNA was synthesized from RNA extracted from 3-day old etiolated Sorghum seedlings using Oligo(dT) priming and SuperScript II Reverse Transcriptase (Invitrogen) according to the manufacturer's instructions. The primers SbMATE-F and SbMATE-R (see Supplementary Table S2) were used to amplify the *SbMATE2* transcript which was cloned into the pCR-Blunt II-TOPO vector using the Zero Blunt TOPO PCR Cloning Kit (Invitrogen). Multiple *SbMATE2* containing plasmids were verified by sequencing to confirm the gene structure. A selected *SbMATE2* clone was used as template to amplify the *SbMATE2* coding region using primers SbMATE-USER-F and SbMATE-USER-R (see Supplementary Table S2), such that it contained the ATG start codon but lacked the stop codon. The fragment was cloned in frame to YFP with USER-based subcloning into pCambia 2300 35S-User_site-YFP (pLIFE0001). Clone pLIFE001_35S-SbMATE2-YFP-(3) was transformed to *Agrobacterium tumefaciencs* AGL1 for use in transient expression studies. Protoplasts from *Nicotiana benthamiana* transiently expressing the SbMATE2-YFP fusion protein were isolated according to Damm & Willmitzer (1988).

Primer SbMATE-USER-F in combination with primer SbMATE-Xenopus-USER-R (see Supplementary Table S2) were used to amplify the *SbMATE2* coding region and insert it in vector pNB1u (pUSER_016), cloning it between the T7 promoter and the 3-UTR region of β-globin (see Nour-Eldin *et al.*, 2006). The construct was verified by sequencing and primers F and R (see Supplementary Table S2) were used to amplify the *in vitro* transcription cassette for producing *SbMATE2* mRNA.

Computational analyses.

The protein structure of SbMATE2 was modelled using the Phyre2 server (http://www.sbg.bio.ic.ac.uk/phyre2) following the procedure outlined in Kelley *et al.* (2015) and the model was visualised using the PyMOL Molecular Graphics System (http://pymol.org/). Phylogenetic analysis was performed using the MEGA5 program suite (Tamura *et al.*, 2011). Clustal Omega (www.ebi.ac.uk) was used for multiple sequence alignment.

Supplementary Figures:

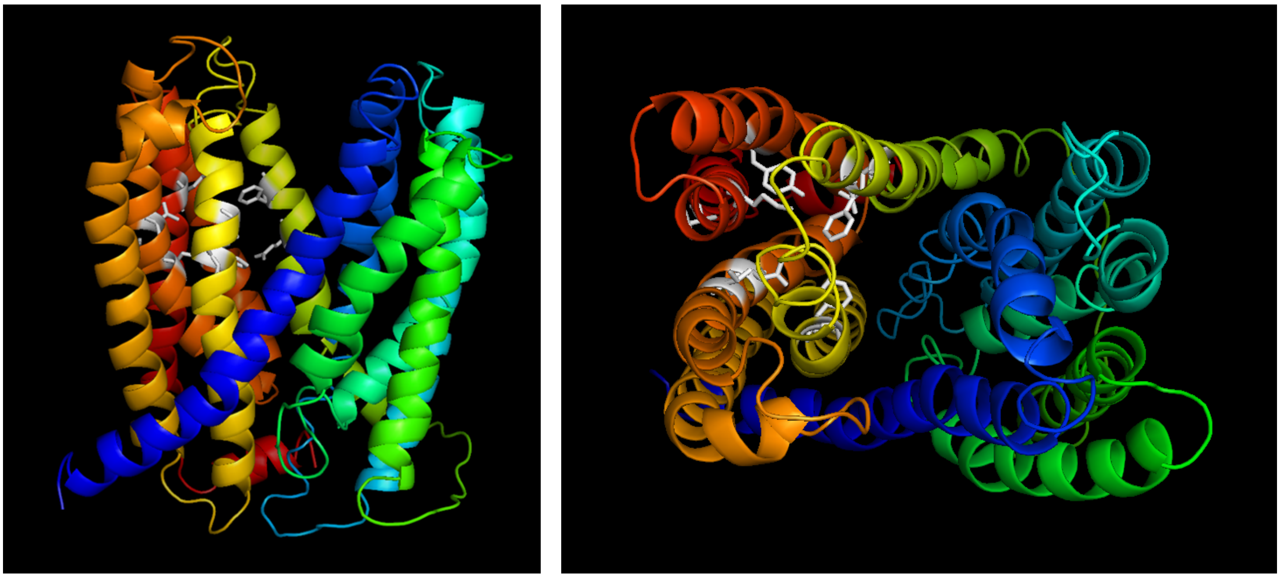

Figure S1: Structural model of SbMate2 predicted using the Phyre2 web portal. The model is based on the structure of the MATE transporter NorM from *Vibrio cholerae* (PDB ID: 3MKU) with 100% confidence over 449 residues (90% of the sequence) (He et al. 2010). (**a)** front view. (**b**) view from the vacuolar lumen side with the individual helices TM1 to TM12 numbered. The amino acid residues in TM8 and TM10 - TM12 that form the by Phyre2 predicted cation binding side are shown as stick structures in white. They are E282, F286, G314, F317, T396, N400, Y427, M453 and T458.

```
Norm-VC     ---------------------------------------------MENSVHRYKKEASNL 15
rbMate1     ---MEA--------PVELG--PGG-RQ--A-------SPERRHWLRCLVLSDFREELRAL 37
Mate1b      ---MER--------TEESAPGPGG-AD--A-------ASE-RRGLRCLLLPGFLEELRAL 38
hMATE1      ---MEA--------PEEPAPVRGG-PE--A-------TLE-VRGSRCLRLSAFREELRAL 38
SbMATE2     ---MDSTTPLLQPAPH----GGGGSRELEAILEDASVP-WARRAL-----RGAGVELPLL 47
TT12        MSSTETYEPLLTRLHSDSQITERSSPEIEEFLRRRGSTVTPRWWL-----KLAVWESKLL 55
                                                           *   *

Norm-VC     IKLATPVLIASVAQTGMGFVDTIMAGGVSAIDMAAVSIAASI-WLPSILFGVGLLMALVP 74
rbMate1     LVLACPAFLAQLMVFLISFVSSVFCGHLSKLELNAVTLAIAVINVMGVSVGFGLSSACDT 97
Mate1b      LVLAGPAFLAQLMMFLISFISSVFCGHLGKLELDAVTLAIAVINVTGISVGHGLSSACDT 98
hMATE1      LVLAGPAFLVQLMVFLISFISSVFCGHLGKLELDAVTLAIAVINVTGVSVGFGLSSACDT 98
SbMATE2     LRIALPAVAVYMINYLMSMSTQIFCGQLGNLELAAVSLGNTGIQVFAYGLMLGMGSAVET 107
TT12        WTLSGASIVVSVLNYMLSFVTVMFTGHLGSLQLAGASIATVGIQGLAYGIMLGMASAVQT 115
              ::   . . :    :.:   :: * :. ::: ..::.       .  .  *:  *

Norm-VC     VVAQLNGAGRQHKIPFFEVHQGLILALLVSVPIIAVLFQTQFIIRFMDVEEAMATKTVGYM 134
rbMate1     LISQTYGSRNLKHVGVILQRGSLILLLCCLPCWALFLNTQHILLLFRQDPAVSRLTQTYV 157
Mate1b      LISQTYGSQNLKHVGVILQRGTLILLLCCFPCWALFINTEQILLLFRQDPDVSRLTQTYV 158
hMATE1      LISQTYGSQNLKHVGVILQRSALVLLLCCFPCWALFLNTQHILLLFRQDPDVSRLTQTYV 158
SbMATE2     LCGQAYGAHKPGMLGVYLQRSTVLLTATGVPLAVAYGFSERILVFLGESERIAHAAAVFV 167
TT12        VCGQAYGARQYSSMGIICQRAMVLHLAAAVFLTFLYWYSGPILKTMGQSVAIAHEGQIFA 175
            : .*  *: .   : .  ::. ::     .        :  *:  :  .  ::     :

Norm-VC     HAVIFAVPAYLLFQALRSFTDGMSLTKPAMVIGFIGLLLNIPLNWIFVYGKFGAPELGGV 194
rbMate1     TIFIPALPATFLYTLQVKYLLNQGIVLPQVVTGVAANLVNALANYLFVYQ----LHLGVM 213
Mate1b      MIFIPALPAAFLYTLQVKYLLNQGIVLPQIMTGIAANLVNALANYVFLYH----LHLGVM 214
hMATE1      TIFIPALPATFLYMLQVKYLLNQGIVLPQIVTGVAANLVNALANYLFLHQ----LHLGVI 214
SbMATE2     YGLIPQIFAYAANFPIQKFLQAQSIVAPSAYISTATLALHLALTWLAVDR----LGMGLL 223
TT12        RGMIPQIYAFALACPMQRFLQAQNIVNPLAYMSLGVFLLHTLLTWLVTNV----LDFGLL 231
              .*  : *         :    .:. *    .     ::   .::          :* :

Norm-VC     GCGVATAIVYWIMLLLLLFYIVTSKRLAHVKVFETFHKPQPKELIRLFRLGFPVAAALFF 254
rbMate1     GSALANTVAQFTLALLLFLYILRSKVYQ--ATWGGWSLECLQDWASFFRLAIPSMLMLCM 271
Mate1b      GSALANTISQFALAIFLFLYILWRRLHQ--ATWGGWSWECLQDWASFLRLAIPSMLMLCI 272
hMATE1      GSALANLISQYTLALLLFLYILGKKLHQ--ATWGGWSLECLQDWASFLRLAIPSMLMLCM 272
SbMATE2     GGALVLSLSWWIIVLAQFGYIVTSPRCR--ETWTGFTSQAFHSLGSFFKLSAASAVMLCL 281
TT12        GAALILSFSWWLLVAVNGMYILMSPNCK--ETWTGFSTRAFRGIWPYFKLTVASAVMLCL 289
            * .:   .  : :      **:         .:  :     :     ::*       * :

Norm-VC     EVTLFAVVALLVAPLGSTVVAAHQV--ALNFSSLVFMFPMSIGAAVSIRVGHKLGEQDTK 312
rbMate1     EWWAYEIGSFLSGILGMVELGAQSV--TYELAVIVYMIPMGLSVAVNVRVGNALGAGNIE 329
Mate1b      EWWAYEVGSFLSGILGMVELGAQSI--TYELAIIVYMIPSGFSVAANVRVGNALGAGNID 330
hMATE1      EWWAYEVGSFLSGILGMVELGAQSI--VYELAIIVYMVPAGFSVAASVRVGNALGAGDME 330
SbMATE2     ETWYFQILVLIAGLLKNPELSLDSLSICMTINGWVFMISVGFNAAASVRVGNELGAGNPR 341
TT12        EIWYNQGLVIISGLLSNPTISLDAISICMYYLNWDMQFMLGLSAAISVRVSNELGAGNPR 349
            *        :: . *    :. . :            .  .:..* .:**.: **  :

Norm-VC     GAAIAANVGLMTGLATACITALLTVLFREQIALLYTENQVVVALAMQLLLFAAIYQCMDA 372
rbMate1     QAKKSSAVALLVTELIAVVFCVMLLSCKDLVGYIFTSDRDIIALVAQVTPIYAVSHLFES 389
Mate1b      QAKKSSAISLIVTELFAVTFCVLLLGCKDLVGYIFTTDRDIVALVAQVIPIYAVSHLFEG 390
hMATE1      QARKSSTVSLLITVLFAVAFSVLLLSCKDHVGYIFTTDRDIINLVAQVVPIYAVSHLFEA 390
SbMATE2     AAAFSVVVVTSLSLAVAVVCAVVVLCIRDQLSYFFTGGEAVARAVSDLCPLLAVTLVLNG 401
TT12        VAMLSVVVVNITTVLISSVLCVIVLVFRVGLSKAFTSDAEVIAAVSDLFPLLAVSIFLNG 409
             *  :  :        :   .:: :  :  :.  :* .  :   . :: :  *:   ::.

Norm-VC     VQVVAAGSLRGYKDMTAIFHRTFISYWVLGLPTGYILGMTNWLTEQPLGAKGFWLGFIIG 432
```

```
rbMate1      LAGTSGGILRGSGNQKFGAIVNAIGYYVVGLPIGIALMFA-----AKLGVIGLWLGIVV- 443
Mate1b       LACTCGGILRGTGNQKVGAIVNAIGYYVIGLPIGIALMFA-----AKLGVIGLWSGIII- 444
hMATE1       LACTSGGVLRGSGNQKVGAIVNTIGYYVVGLPIGIALMFA-----TTLGVMGLWSGIII- 444
SbMATE2      VQPVLSGVAVGCGWQAFVAYVNVGCYYIIGVPLGVFLGFY-----LDLGAKGIWSGMVIG 456
TT12         IQPILSGVAIGSGWQAVVAYVNLVTYYVIGLPIGCVLGFK-----TSLGVAGIWWGMIA- 463
             :    .*   *          .   *:::*:* *  * :        **. *:* *::

Norm-VC      LSAA----ALMLGQRLYWLQKQSDDVQLHLAAK--------------------------- 461
rbMate1      CAVSQAVCFLGFIARLNWTKACQ-QARVHANLTVNTAS-NGNSAVLPDQPHPVGPDSHGG 501
Mate1b       CTTCQTTCFLAFIARLNWKRACQ-QAQVHANLKVNVAL---NSAVSHEPAHPVCPESHGE 500
hMATE1       CTVFQAVCFLGFIIQLNWKKACQ-QAQVHANLKVNNVPRSGNSALPQDPLHPGCPENLEG 503
SbMATE2      GTMMQTLILLWVTSRTDWNKEVE-KARARLDKWDDKKQPLLED----------------- 498
TT12         GVILQTLTLIVLTLKTNWTSEVE-NAAQRVKTSATENQEMANAGV--------------- 507
                      : .  :  *    . ..  :

Norm-VC      ------------------------------------------------------------ 461
rbMate1      IVLRDADRKEGAELNEQVHPELPLPVRPEDSAHLSGKQLALRRGLLLLGVILVLLAGILV 561
Mate1b       IMMTDLEKKDETQLDQPMNQQQALPIRPKDSNKLSGKQLALRRGLLLLGVVLVLVGGILV 560
hMATE1       ILTNDVGKTGEPQSDQQMRQEEPLPEHPQDGAKLSRKQLVLRRGLLLLGVFLILLVGILV 563
SbMATE2      ------------------------------------------------------------ 498
TT12         ------------------------------------------------------------ 507


Norm-VC      -------      460
rbMate1      KVYVRTQ      568
Mate1b       RVYIRIE      567
hMATE1       RFYVRIQ      570
SbMATE2      -------      498
TT12         -------      507
```

Figure S2: Multiple sequence alignment of MATE transporters from prokaryotic and eukaryotic origin. The alignment was generated with Clustal Omega (www.ebi.ac.uk). The amino acid sequences were obtained from genbank and phytozome and include: NorM-VC from *Vibrio cholera* (Genbank accession: 3MKU_B), rbMATE1 from *Oryctolagus cuniculus* (NP_001103289), Mate1b from *Mus musculus* (NP_080459), hMATE1 from *Homo sapiens* (AAH10661), SbMATE2 from *Sorghum bicolor* (Sobic.001G012600), and TT12 from *Arabidopsis thaliana* (Q9LYT3, At3G59030). Conserved amino acids that form a cation-binding site are highlighted in yellow. Most notably these are in SbMATE2 E282 (E255 in NorM-VC), F286 (F259), F317 (F288), N400 (D371), and Y427 (Y398). See also Figure 4 in He *et al.* 2010.

Supplementary Tables

Table S1: ***Sorghum bicolor*** **publically available RNA-Seq expression data present in the MOROKOSHI database with references to the original literature** (http://sorghum.riken.jp/morokoshi/)

Sample number	Sample name	Tissue and treatment	Reference
**1**	Spikelet	at anthesis, 5 months after sowing	Makita *et al.*, 2014
**2**	Seed	5 months + 2 weeks after sowing	
**3**	Stem	5 months after sowing	
**4**	Infec_C0h	Control, 0 hours	Yazawa *et al.*, 2013
**5**	Infec_C12h	Control, 12 hours	
**6**	Infec_12h	Infected, 12 hours	
**7**	Infec_C24h	Control, 24 hours	
**8**	Infec_24h	Infected, 24 hours	
**9**	Nitrogen	Root tissue, nitrogen stress	Gelli *et al.*, 2014
**10**	Root_ABA	Roots 9 day seedlings treated with 20 µM ABA	Dugas *et al.*, 2011
**11**	Root_PEG	Roots 9 day seedl. treated with 20% PEG-8000	
**12**	Root_seedling	Roots 9 day seedlings, untreated	
**13**	Root_NaOH	Roots 9 day seedlings, treated 0.2 M NaOH	
**14**	Shoot_ABA	Shoots 9 day seedl. treated with 20 µM ABA	
**15**	Shoot_PEG	Shoots 9 day seedl. treated with 20% PEG-8000	
**16**	Shoot_seedling	Shoots 9 day seedlings, untreated	
**17**	Shoot_NaOH	Shoots 9 day seedlings, treated 0.2 M NaOH	
**18**	Leaves	BTx623, 20 day leaves	Davidson *et al.,* 2012
**19**	Inflorescence_emerg	BTx623, emerging inflorescence	
**20**	Seed_5d_pollination	BTx623, seed 5 days after pollination	
**21**	Inflorescence_early	BTx623, early inflorescence	
**22**	Pistil	BTx623, pistil	
**23**	Embryo	BTx623, embryo 25 days after pollination	
**24**	Endosperm	BTx623, endosperm 25 days after pollination	
**25**	Anther	BTx623, anther	
**26**	Seed_10d_pollination	BTx623, seed 10 days after pollination	

Supplementary Table S2
Sequences of primers used in this study

Primers for cloning the *SbMATE2* (Sobic.001G012600) cDNA clone
SbMATE-F: 5'-CGATCGATCAACGTGCTAGCTTTGC-3'
SbMATE-R: 5'-AGTCTTGTTCTAAATGATGATGGCAG-3'

Primers for USER-based subcloning *SbMATE2* into pCambia 2300 35S-USER_site-YFP (pLIFE0001)
SbMATE-USER-F 5'-GGCTTAAUATGGACTCGACGACGCCGCT-3'
SbMATE-USER-R: 5'-GGTTTAAUCCGTCTTCTAGGAGAGGCTGCTTCTTATCATCCCACT-3'

Primers for cloning into T7 promoter vector pUSER_016
SbMATE-USER-F 5'-GGCTTAAUATGGACTCGACGACGCCGCT-3'
SbMATE-Xenopus-USER-R
5'-GGTTTAAUTCAAGCGTAATCTGGAACATCGTATGGGTAGTCTTCTAGGAGAGGCTGCTTCTTATCATCCCACT-3'

Primers for amplification of *in vitro* transcription cassette for producing *SbMATE2* mRNA
F: 5'-TGCAAGGCGATTAAGTTGGGTAACGC -3'
R: 5'-CTCGAGGCGGCCGCCTGCAG-3'

Supplementary references:

Damm, B. & Willmitzer, L. Regeneration of fertile plants from protoplasts of different *Arabidopsis thaliana* genotypes. *Mol. Gen. Genet.* **213,** 15-20 (1988).

Davidson, R.M. *et al.* Comparative transcriptomics of three Poaceae species reveals patterns of gene expression evolution. *Plant J.* **71,** 492-502 (2012).

Dugas, D.V. *et al.* Functional annotation of the transcriptome of *Sorghum bicolor* in response to osmotic stress and abscisic acid. *BMC Genomics* **12,** 514 (2011).

Gelli, M. *et al.* Identification of differentially expressed genes between sorghum genotypes with contrasting nitrogen stress tolerance by genome-wide transcriptional profiling. *BMC Genomics* **15,** 179 (2014).

He, X. *et al*. Structure of a cation-bound multidrug and toxic compound extrusion transporter. *Nature* **467,** 991-996 (2010).

Kelley, L.A. *et al.* The Phyre2 web portal for protein modeling, prediction and analysis. *Nat. Protoc.* **10,** 845-858 (2015).

Makita, Y. *et al*. MOROKOSHI: transcriptome database in *Sorghum bicolor*. *Plant Cell Physiol.* **56,** e6 (2015).

Nelson, B.K., Cai, X &, Nebenführ A. A multicolored set of in vivo organelle markers for co-localization studies in Arabidopsis and other plants. *Plant J*. **51,** 1126-1136 (2007).

Tamura, K. *et al.* MEGA5: Molecular evolutionary genetics analysis using maximum likelihood, evolutionary distance, and maximum parsimony methods. *Mol. Biol. Evol.* **28,** 2731-2739 (2011).

Yazawa, T., Kawahigashi, H., Matsumoto, T. & Mizuno, H. Simultaneous transcriptome analysis of sorghum and *Bipolaris sorghicola* by using RNA-seq in combination with *de novo* transcriptome assembly. *Plos One* **8,** e62460 (2013).
